# Using the Reproducible Open Coding Kit & Epistemic Network Analysis to model qualitative data

**DOI:** 10.1080/21642850.2022.2119144

**Published:** 2022-12-28

**Authors:** Szilvia Zörgő, Gjalt-Jorn Peters

**Affiliations:** a Care and Public Health Research Institute, Maastricht University, Maastricht, the Netherlands; b Department of Methodology & Statistics, Faculty of Psychology, Open University, Heerlen, the Netherlands

**Keywords:** Methodology, tutorial, Epistemic Network Analysis (ENA), Reproducible Open Coding Kit (ROCK), data visualization

## Abstract

**Background:** Epistemic Network Analysis (ENA) is a unified, quantitative – qualitative method aiming to draw from both methodological worlds by leveraging a data set containing raw and quantified qualitative data, as well as metadata about data providers or the data itself. ENA generates network models depicting the relative frequencies of co-occurrences for each unique pair of codes in designated segments of qualitative data.

**Methods:** This step-by-step tutorial demonstrates how to model qualitative data with ENA through its quantification via coding and segmentation. Data was curated with the Reproducible Open Coding Kit (ROCK), a human- and machine-readable standard for representing coded qualitative data, enabling researchers to document their workflow, as well as organize their data in a format that is agnostic to software of any kind.

**Results:** ENA allows researchers to obtain insights otherwise unavailable by depicting relative code frequencies and co-occurrence patterns, facilitating a comparison of those patterns between groups and individual data providers.

**Conclusions:** ENA aids reflexivity, moves beyond code frequencies to depict their interactions, allows researchers to easily create post-hoc groupings of data providers for various comparisons, and enables conveying complex results in a visualization that caters to both qualitative and quantitative sensibilities.

## Introduction

### Where the twain shall meet

The distinction between quantitative and qualitative research methodologies can, for some, manifest as a hierarchy in the reliability of results: the “hard facts” from quantitative research versus the “soft opinions” from qualitative initiatives. Many scholars have examined other dichotomies attributed to these methodologies, such as “objective” versus “subjective”, “masculine” versus “feminine” (Gherardi & Turner, 2002). From a utilitarian epistemological perspective, such dichotomization is unhelpful: instead, different methods serve different research purposes. Quantitative methods enable estimating parameters, applying inferential methods, and obtaining dichotomous answers (which serves various epistemic and pragmatic purposes in its own right; Uygun Tunç et al., 2021). However, quantitative methods require measurement of the relevant phenomena, and this is not always possible or desirable. Qualitative methods, on the other hand, enable studying phenomena even when almost nothing is known about them yet, instead facilitating incremental development of that understanding. This makes them suitable for answering research questions regarding the depth of partially understood/mapped phenomena, how these manifest individually, or exploring completely new phenomena.

Quantitative and qualitative methods are sometimes mixed within a study and take on complementary functions. This complementary relationship can exist regarding the research questions (e.g., using a survey to answer one question and interviews to answer another) or as methodological triangulation to answer the same research question from different methodological “angles” (e.g., “confirming” results of focus group discussions with a survey or “enriching” questionnaires with open-ended questions). Most frequently, using mixed methods denotes collecting different data with each employed method, and thus juxtaposing findings is often troublesome (Tariq & Woodman, 2013). Not only do quantitative and qualitative methods typically serve different research purposes, prohibiting straightforward comparisons of the respective methods’ results, their sampling considerations also usually vary a great deal. One of the strengths of quantitative methods is the ability to model error terms, which requires random sampling and systematic data collection (e.g., using questionnaires, response latencies, or other standardized paradigms). Conversely, one of the strengths of qualitative methods is often the ability to adapt data collection based on the rich data and real-time insights. These data are expensive to collect and analyze, and sampling is therefore often purposive (e.g., to obtain heterogeneous samples) and data collection is typically non-systematic (e.g., interviews or naturally occurring data such as interactions on social media).

Epistemic Network Analysis (ENA) exemplifies a unified, quantitative – qualitative method aiming to draw from both methodological worlds with a data set containing raw and quantified qualitative data, as well as metadata about data providers and the data itself. ENA generates network graphs from such quantified qualitative data; it depicts the relative co-occurrence frequencies of codes in designated segments of qualitative data (Williamson Shaffer et al., 2016). This is a step-by-step tutorial demonstrating how qualitative data can be quantified through coding and segmentation and subsequently modeled with ENA. To prepare our data consistent with Open Science principles (UNESCO Recommendation on Open Science, 2021), we will use the Reproducible Open Coding Kit (ROCK), a human- and machine-readable standard for representing coded qualitative data, enabling researchers to organize their data, document their workflow, and exchange data in a format that is agnostic to software of any kind. As yet, the ROCK standard is implemented in two pieces of open-source software: an R package called rock and a rudimentary, browser-based graphical user interface called the interface for the Reproducible Open Coding Kit (iROCK). We will be using both of these throughout the tutorial. Snippets of data and a curated data set will be available as a resource for readers to follow along and try various analyses in an RStudio Cloud project accessible with an RStudio Cloud account here: https://rock.science/2hr-workshop.

In the following, we describe the research from which we obtained the data for the tutorial, provide an overview of code development and application, as well as offer some example results to illustrate the potential of epistemic networks. Following this, in the “Step-by-step tutorial” section, we elaborate the ROCK standard, which plays a crucial role in data curation and then expound the process of coding and segmentation. Finally, we explain how epistemic networks are constructed and discuss some strengths and limitations of the technique.

## Information on worked example

### Research question and data collection

The study providing the worked example in this tutorial was aimed at exploring cognitive and behavioral patterns in patient decision-making via semi-structured interviews. We were interested in why patients in four illness groups choose biomedical treatments versus Complementary and Alternative Medicine (CAM). Data from 26 participants was collected in Budapest, Hungary in 2019; patients were recruited via convenience, non-proportional quota sampling to maximize sample heterogeneity, stratifying on therapy choice (Biomedical and CAM), primary diagnosis (D1: Diabetes /I, II, pre-diabetes/, D2: Musculoskeletal diseases, D3: Digestive illnesses, D4: Nervous system diseases), and sex (males and females). Interviews were conducted by four researchers trained in qualitative methods, the employed interview structure, and the code system. Interviews lasted 60 minutes on average, ranging from 40 to 120 minutes; they were sound-recorded and transcribed verbatim. Table 1 shows the main topics of the semi-structured interview, its subtopics, and the number of questions pertaining to these. For a full description of data collection and results, see: Zörgő, Swiecki, et al., 2021; Zörgő & Peters, 2019.

**Table 1.** Areas of the semi-structured interview and number of related questions.

Parent code	Content	Question load
Epistemology	**Information** sources of health-related information, appraisal of information	6 questions + probes
Ontology	**Explanatory Model** concepts of illness and health, etiology	4 questions + probes
Behavior	**Patient journey** choices of therapy, evaluation of therapeutic efficacy	5 questions + probes

For the purposes of this tutorial, we focus on one research question: Are there any differences between how biomedicine versus CAM users conceptualize etiology of illness, and if so, what are these differences? The answer to this research question aims to contribute to an understanding of why patients employ conventional or non-conventional therapies. We will be investigating factors in decision-making in the biomedicine versus CAM subsamples (13 participants each), and also in smaller groups based on diagnosis.

### Code development and employed codes

The research question in our worked example will be addressed with a set of codes pertaining to the patients’ etiology. These codes were developed inductively based on a fully qualitative project on patient decision-making regarding therapy choice carried out between January 2015 and June 2017 in Hungary. Our code system is founded on this previous study (Zörgő et al., 2018); we adopted that code system and applied the codes deductively to our novel narrative corpus. It is comprised of three levels of abstraction, containing 52 low-level codes in total. Codes employed in this tutorial correspond to the “Ontology” section of our data collection tool (Table 1) and are listed in Table 2.

**Table 2.** Codes employed in the tutorial and their short description.

Grandparent code label	Parent code label	Child code label	Code identifier	Code description
**Ontology**	**Etiology**	Psychosocial	Psych	Emotion, stress, trauma, nerves (no mention of energy)
		Vitalistic	Vital	Energy/qi/prana, flow, block, law of attraction, nothing is by chance
		Ecological	Eco	Environmental toxins, chemicals, “electro-smog”
		Nutritional	Nutri	Quality or type of food, additives
		Genetic	Genetic	Inherited illness or susceptibility, genes, “runs in the family”

### Example results

**Figure 1.**
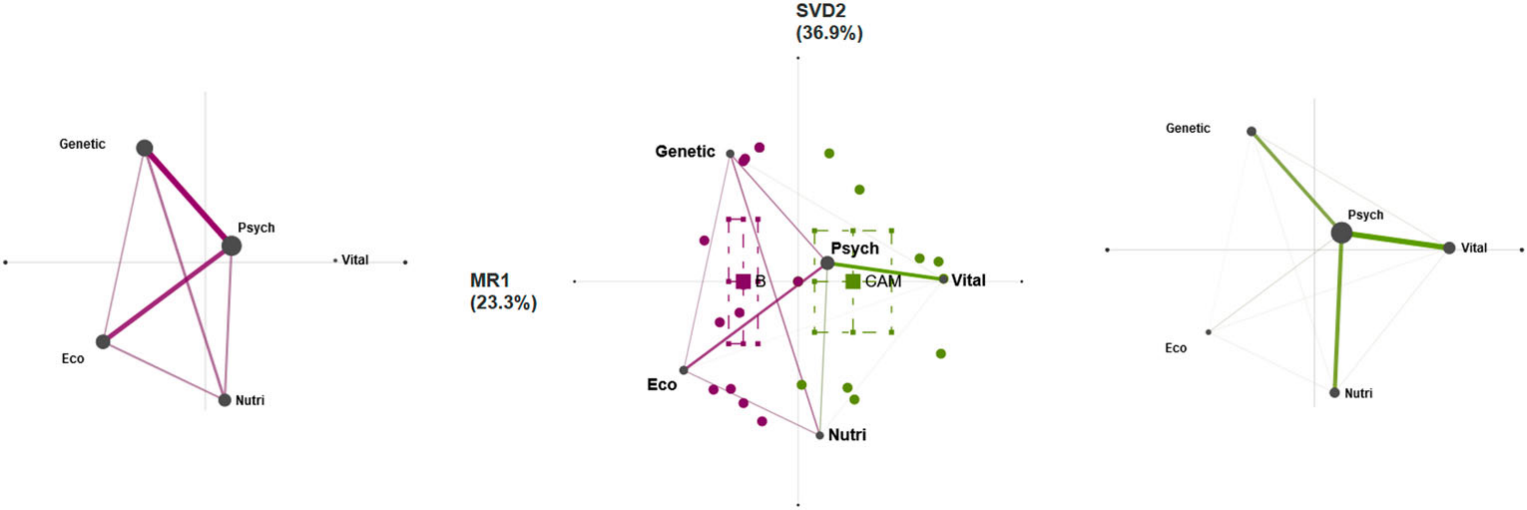
Mean epistemic networks for the biomedicine group (purple, left) and the CAM group (green, right) and a comparison plot (center) showing the differences between mean networks.

To demonstrate the utility and possibilities of jointly employing the ROCK and ENA, we first briefly describe our results. We explored differences between narratives from biomedicine versus CAM users regarding their etiology of illness. Figure 1 contains mean epistemic networks for the biomedicine group (purple, left) and the CAM group (green, right) showing the co-occurrences among etiology codes in given segments of data. The thickness and saturation of the edges (lines) indicate the relative frequency of co-occurrence between each pair of codes; the size of the nodes (black circles) indicates the relative frequency of each code within that group. Each code’s (node’s) position in the two-dimensional space is determined by the results of the sequential application of two data-reduction techniques to the cumulative matrix containing code co-occurrences. The x axis represents the results of the technique applied first, means rotation (see below), which produces a single component such that the difference between the two groups is maximized. The y axis represents the results of the second technique, singular value decomposition, applied to the residual co-occurrence counts after subtraction of the co-occurrence count computed from the means rotation.

In the center is a difference graph (comparison plot) showing the subtracted mean networks of the biomedicine group and the CAM group. The thickness and saturation of each line indicates the relative difference between the two groups: purple lines indicate connections with higher relative frequencies among biomedicine users, and green lines indicate connections with higher relative frequencies among CAM users. The points show the network locations (ENA scores) of each biomedicine user (purple points) and CAM user (green points). The colored squares are the mean network locations (mean ENA scores) of each group, and the dashed lines around the means represent the 95% confidence intervals on each dimension.

As seen in the thickness of the edges in Figure 1 on the left, Biomedicine users are more prone to simultaneously discuss psychosocial, genetic, and ecological factors as coded within illness etiology, while CAM user narratives (fig. 1, right) show a more frequent co-occurrence of psychosocial, vitalistic, and nutritional factors. The difference graph highlights the key difference between the two subsamples: CAM users’ expressions were frequently simultaneously coded with psychosocial and vitalistic factors, while the latter factor never co-occurred with any code in the biomedicine sample.

**Figure 2.**
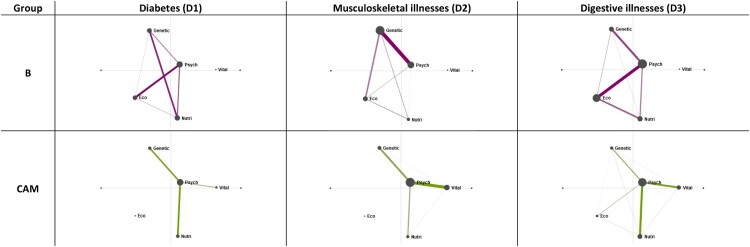
Mean epistemic networks for biomedicine (purple, top row) and CAM users (green, bottom row) according to diagnosis groups (D1-3).

Network graphs can also be created to answer the second part of our research question: can we see differences in etiologies among the various diagnosis groups? Below are network models depicting Biomedicine versus CAM user narratives based on diagnosis group (D1-3) Figure 2.

We can visually inspect differences in all these models and arrive at conclusions such as: codes for psychosocial and vitalistic factors most often co-occur in the D2 CAM group and least often in the D1 CAM group. Thus, in our diabetes subsample, vitalism does not play a crucial constitutive role in etiology; in this subgroup the presence or absence of ecological factors within etiology may explain differences more, apart from belief in vitalism, which is a key difference between the Biomedicine and CAM group in general (as seen in fig. 1).

The displayed networks and their structure of connections can be further examined both quantitatively and qualitatively. Edge weights are also quantified (falling between 0 and 1), capturing the relative frequency of co-occurrence between two codes in given segments of data. These connections can also be examined qualitatively by using the rock R package or the ENA webtool to go back to the coded narratives and interpret the co-occurrence of codes via de- and re-contextualization. Because all models generated above share a projection space and are thus coordinated representations of our data, the location of nodes in all networks is identical and the location of individual units (ENA scores of patient networks) is meaningful: ENA scores in the vicinity of certain codes represent narratives that are more saturated with co-occurrences between those codes, and similarly, the location of nodes in relation to each other (how the nodes of a network appear in the projection space) carries information about their co-occurrences in the corpus.

Below we disclose how these networks were generated by discussing the ROCK standard, clarifying relevant terms used in ENA, and describing a step-by-step account of data curation, coding and segmentation, and model parameterization. We will conclude by emphasizing the advantages and limitations of employing this method to model qualitative data.

## Step-by-step tutorial

### Creating a project

This tutorial does not discuss any methodological aspects of general research design or data collection; it begins at the point where all necessary data has been collected. To organize the data and transparently document changes, we recommend using a specific directory structure that corresponds to the workflow we will explain in this tutorial. To facilitate starting new projects with this workflow, we will use the Clean Lean Initial Formatted File (CLIFF) (https://rock.science/cliff). The CLIFF is an R script comprising all essential commands from the rock R package and its repository also contains a basic directory structure that can be used when running a ROCK project. Once downloaded, the CLIFF can be tailored to a specific project.

The CLIFF repository contains three main directories: data, results, and scripts. This structure was designed to accommodate a clear workflow, where data at each stage in the qualitative project is housed in separate directories. This enables the researcher to keep track of changes to their data, to be able to pinpoint any glitches in processes and easily correct them, or to create different versions of analyses within the project branching from any given stage. The file structure used in the CLIFF will be referenced and explained in different sections of this tutorial.

### The ROCK standard and examples for its operationalization

#### Sources and utterances

**Figure 3.**
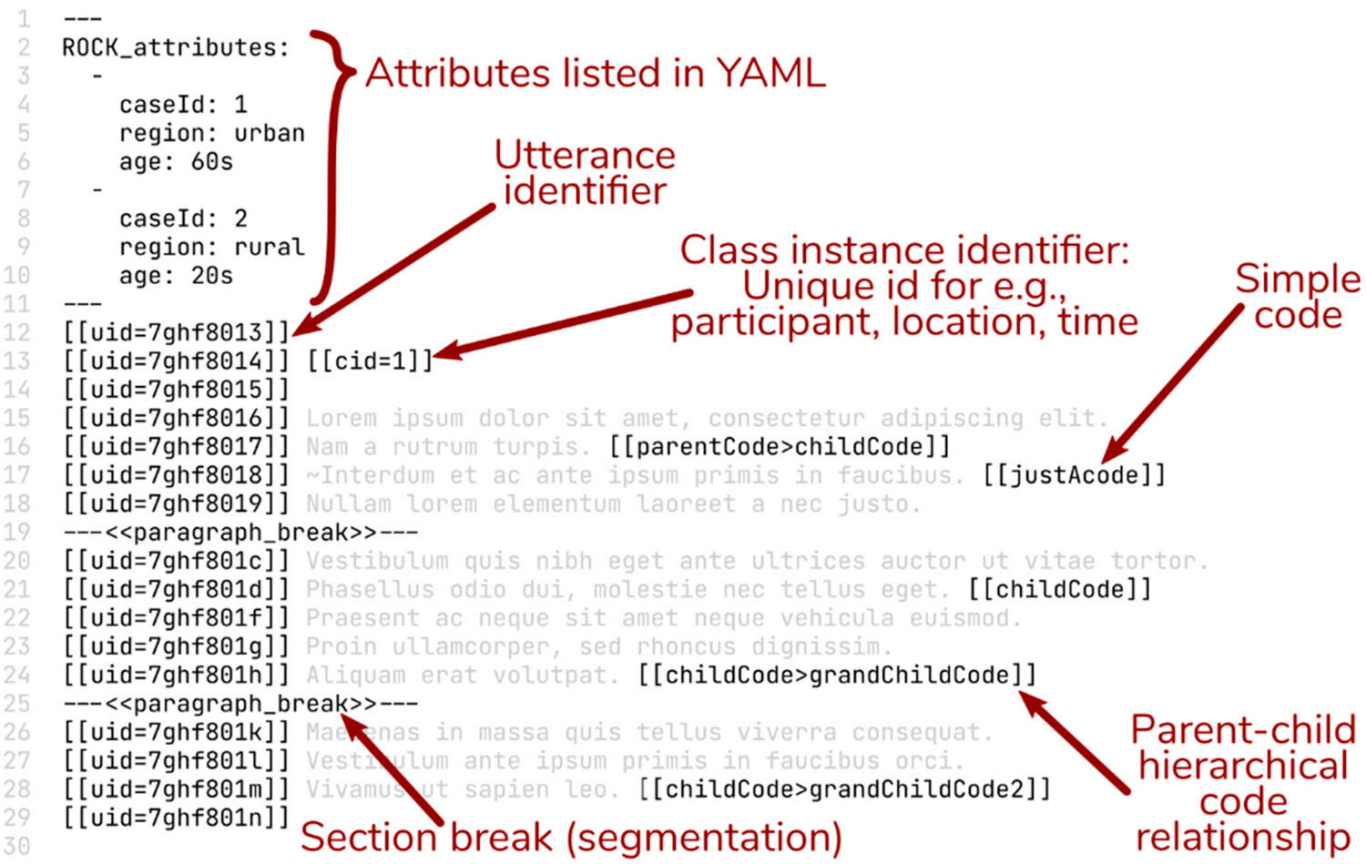
A fictional annotated data source that has been coded with the Reproducible Open Coding Kit.

In the ROCK standard, data are organized into sources: plain text files with the extension .rock. Sources can be, for example, transcripts of interviews or focus group discussions, log files, legal documents, or social media threads. An example of a fictitious coded source is provided in Figure 3. Sources contain one or more utterances; this term is used to denote the smallest codable segment in qualitative data (as per the researchers’ decision). Examples of utterances include sentences in an interview transcript, turns-of-talk in a discussion, paragraphs in legal documents, and posts and replies in threaded data. The term utterance can also be operationalized as a second (or any duration) in time or an action in a series of behaviors; it is a flexible concept to accommodate a wide range of qualitative data one may work with. In version 1.0 of the ROCK standard, codes are always applied at the utterance level, making this level of segmentation mandatory. For the purposes of our tutorial, each source holds one interview transcript, and within each source, we have operationalized utterances as sentences. It is worth noting that this definition of an utterance, ultimately operationalized in terms of punctuation, causes the process of transcription to become a key form of data transformation, where the decisions made (e.g., identifying which words should be grouped into sentences, adding punctuation) have an effect on later processes. Had we operationalized utterances differently (e.g., “strings of ten consecutive words”), that operationalization would also have had effects, albeit different ones.

#### Identifiers

The next important facet of the ROCK standard are identifiers, which are unique text strings used to encode abstract concepts in a way that is simultaneously machine-readable and human-readable. For example, every code has a unique code identifier; this is discussed more in detail below. Similarly, extraneous information about data can be attached by encoding class instance identifiers. Classes can be, for example, data providers (e.g., participants), coders, times of day, or data collection sites. Class instance identifiers are used to label specific instances of classes, such as individuals providing or coding data. A frequently applied class instance identifier is a case identifier, which helps specify data providers (e.g., interviewees); another common identifier specifies the researcher who collected data (e.g., interviewer) or who coded data (i.e., coder). Class instance identifiers consist of the class identifier followed by an equals sign and the identifier of the instance within that class, all between two pairs of square brackets. In this tutorial we highlight the classes and class instance identifiers listed in Table 3 (also see Figure 3).

**Table 3.** Classes, their operationalization within the tutorial, and examples for class instance identifiers.

Class	Operationalization	Class instance identifier examples
Utterance	Sentence	[[uid = 756g3sy2]], [[uid = 756g3sy3]]
Case	Individual interviewee	[[cid = 1]], [[cid = 2]]
Coder	Individual coder	[[coderId = Jenna]], [[coderId = Chris]]

An utterance identifier (uid) can be easily designated with the rock R package, and is a unique combination of letters and numbers that make individual utterances identifiable. In our example, since our lowest level of segmentation (utterance) was operationalized as a sentence, each sentence in all of our interviews receives a unique utterance identifier. Utterances are separated by an utterance marker, which in the ROCK standard is defined as a newline character. This results in each utterance inhabiting its own line in a source (also see section: “Prepending uids”).

A case is a data provider (e.g., a participant, a group, an institution), and so a source contains data from one or more cases. For instance, a focus group discussion or a thread of social media data contains data from multiple cases. In our example, because our interviews were conducted with individual participants, each of our sources contains data from a single case. As part of data curation, we may want to assign specific utterances to specific cases, which we can do by adding the corresponding class instance identifier. We could assign individual utterances to cases by placing a [[cid = 1]] on each utterance’s line, but the ROCK also accommodates “persistent identifiers”. Once designated within a source, this type of identifier will also be applied to each succeeding utterance until a new persistent identifier of the same class (e.g., a new case identifier) is designated.

In our project, since all our sources contain data from single cases, we placed the case identifier of the data provider on the first line of the source, which allows us to parse this source as it applies to all subsequent lines of data within the source. If we were working with focus group transcripts, for example, we would specify the case identifier of each data provider beside their first utterance each time they switch turns in talking. We configured coder identifiers as persistent identifiers, too, because there were multiple coders collaborating in our project, and each coder had a different set of codes that they applied to the data.

#### Attributes

Attributes are used to encode contextual information and other characteristics of the data collection. For example, sources may have attributes such as whether an interview was conducted pre- or post-intervention and cases may have attributes like clinical or sociodemographic data. Examples of our attributes include: interviewee sex, age, level of education, diagnosis type (D1-4), specific illness, illness onset, time of diagnosis, and therapy choice (biomedicine or CAM). Once data are curated, attributes can be used to group utterances and make various comparisons and analyses. In the ROCK standard, attributes are designated in a specific format and can be added to specific sources or listed jointly in a separate plain text file created for this purpose (see section: “Specifying attributes”).

#### Codes

Codes in qualitative research represent constructs, terms, expressions, concepts, or ideas relevant to the research question(s). Codes are identified and indicated in the data. In the ROCK standard, codes are attached to an utterance using the corresponding code identifiers placed between two pairs of square brackets (e.g., [[CodeName]]). If one is using a hierarchical code structure, that hierarchy can be indicated in the code label with a greater-than sign (e.g., [[Parent > Child]] or [[Fruit > Apple]]). In this tutorial, we use [[Etiology > Psych]], [[Etiology > Vital]], [[Etiology > Eco]], [[Etiology > Nutri]], and [[Etiology > Genetic]]. Codes can be developed and applied inductively (grounded, data-driven coding) or adopted and applied deductively (top-down, theory-driven); a combination of these can also be employed. The ROCK can accommodate both types of coding processes (see section: “Manual coding & segmentation with iROCK”).

#### Segmentation

Segmentation is the act of dividing data into segments based on some meaningful procedure. By operationalizing utterances, the smallest codable units within data, we have taken the initial step in segmentation. ENA requires segmentation at higher levels, too: the length of a meaningful segment in the data will determine which codes can co-occur with each other in that segment. This higher level of segmentation can be referred to as a “stanza”; a stanza is a set of one or more utterances that are contextually related to each other. The ROCK standard allows for many types of higher-level segmentation through the use of section breaks, designated by section break identifiers. Section breaks occur between two utterances, separated from those utterances by newline characters. Unlike other identifiers, they are not delimited with double brackets, but with three dashes and sets of two mirrored smaller-than and greater-than signs. For example, valid section breaks are: --- << newQuestion >> --- and --- << stanza_delimiter >> ---. In our tutorial, we will be using stanzas defined as a topic within the semi-structured interview, based on the interview guide. Table 4 contains definitions and examples for discussed elements of the ROCK standard.

**Table 4.** Elements of the ROCK standard.

Standard	Brief description	Examples
Source	Plain text files containing (to be) coded qualitative data	Interviews or focus group transcripts, log files, legal documents, social media threads
Utterance	Smallest codable segment in data; lowest level of segmentation	Sentences in an interview transcript, a turn-of-talk in a discussion, paragraphs in legal documents, posts and replies in threaded data, a second in time
Identifier	Text strings used to encode concepts (code identifiers; also see Code); or class instances (class instance identifiers) to record extraneous information about data	Class instance identifiers: case identifier (to specify data provider), coder identifier (to specify which researcher coded data), utterance identifier (to label smallest codable segments of data), code identifiers (to label utterances with codes)
Attribute	Variables holding contextual information, characteristics of data collection or data providers, associated to data through class instance identifiers	Source attributes: e.g., date of interview; case attributes: e.g., clinical and sociodemographic data
Code	Constructs, terms, expressions, ideas relevant to research questions, associated to data through code identifiers	Psychosocial etiology, Vitalistic etiology, Genetic etiology
Segmentation	Dividing data into meaningful segments by indicating the smallest codable segments and groupings of those; segmentation can be hierarchical	Utterance (lowest level of segmentation, e.g.: a sentence, a response), stanza (set of one or more utterances, e.g.: a response to a question, a topic in a response)

### Organizing and curating data

We now refer back to the CLIFF, which is basically an example script for a project that uses the ROCK standard and the rock R package accompanied by a potential directory structure. All commands can be run directly from the script by e.g., opening the R project in RStudio, opening the script file, and clicking on “run current chunk” (green play button). As a step zero, be sure to run the first chunk in the script entitled “cliff-setup”.

#### Cleaning sources

Within the data directory, the folder called “010—raw-sources” is designated for the original qualitative data transcribed into plain text files. Since data is usually messy right after transcription, it may need some cleaning; for example, the default settings in the rock package for cleaning sources include replacing double periods with single ones and replacing four or more periods with an ellipsis. The full list of defaults can be accessed by typing ?rock::clean_sources into the R Studio console, and can be specified according to the needs of the data. The command for cleaning sources with the rock package is: rock::clean_sources(). The full command is in the CLIFF in the R chunk labeled “cliff-data-preparation-cleaning”. The input and output locations have been pre-specified but can be modified; by default, the cleaned files will be written to the “020—cleaned-sources” directory. A crucial facet of cleaning sources is that each utterance (defined as sentences by default) will be separated by a newline character (i.e., placed on a new line).

#### Prepending utterance identifiers

Next, we designate utterance identifiers for each utterance. The command for achieving this is: rock::prepend_ids_to_sources(); the cleaned files with prepended utterance identifiers will be written to the “030—sources-with-UIDs” directory. To distinguish sources in this stage from sources earlier or later in the data curation process, we suggest specifying a suffix to append (a so-called “slug”, e.g., _withUIDs) that will be added to the source file names. Designating a unique identifier for each utterance in your data set will allow you to refer unequivocally to specific utterances and thus compare and merge coded sources from multiple coders. Table 5 illustrates a paragraph of text from a raw source and its cleaned version with utterance identifiers assigned to each line.

**Table 5.** A paragraph of text (left) and the cleaned version with utterance identifiers (right).

Raw source example	Cleaned source with utterance identifiers
Lorem ipsum dolor sit amet, consectetur adipiscing elit. Quisque pellentesque finibus varius. Nulla et elementum urna. Nunc varius quam eros, vel semper justo venenatis ac. Ut vulputate nunc ac metus egestas ullamcorper. Pellentesque dictum vehicula justo, quis pharetra ante convallis ac. Proin vulputate odio eget augue tincidunt tempus. Curabitur ullamcorper nunc et risus gravida, imperdiet blandit turpis commodo. Maecenas in eros egestas, auctor felis nec, interdum velit.	[[uid = 7jlh4gbt]] Lorem ipsum dolor sit amet, consectetur adipiscing elit. [[uid = 7jlh4gbw]] Quisque pellentesque finibus varius. [[uid = 7jlh4gbx]] Nulla et elementum urna. [[uid = 7jlh4gby]] Nunc varius quam eros, vel semper justo venenatis ac. [[uid = 7jlh4gbz]] Ut vulputate nunc ac metus egestas ullamcorper. [[uid = 7jlh4gc0]] Pellentesque dictum vehicula justo, quis pharetra ante convallis ac. [[uid = 7jlh4gc1]] Proin vulputate odio eget augue tincidunt tempus. [[uid = 7jlh4gc2]] Curabitur ullamcorper nunc et risus gravida, imperdiet blandit turpis commodo. [[uid = 7jlh4gc3]] Maecenas in eros egestas, auctor felis nec, interdum velit.

#### Specifying persistent identifiers and section breaks

Below is an example of an R chunk in the CLIFF where a number of rock package options are configured. In this example, we have specified the persistent identifier in our project (case id) and our chosen form of higher-level segmentation (stanza).

**Table:** 

rock::opts$set( persistentIds = “caseId”, sectionRegexes = c(StanzaDelimiter = “--- << stanza_delimiter >> ---”), silent = TRUE );

By doing so, we can now manually add our case identifiers (e.g., [[cid = 1]]^1^) to the beginning of our sources when we code them; this will let the ROCK know that every utterance in that source came from the same case. (If there are multiple cases per source, case identifiers need to be designated at every turn-of-talk.) By specifying our chosen section break(s), we are preparing for coding and segmentation (see below). When in a later phase we will perform higher level segmentation in our narratives, it will be possible to aggregate utterances based on this section break and their sections will be numbered automatically in our data frame.

#### Specifying attributes

**Figure 4.**
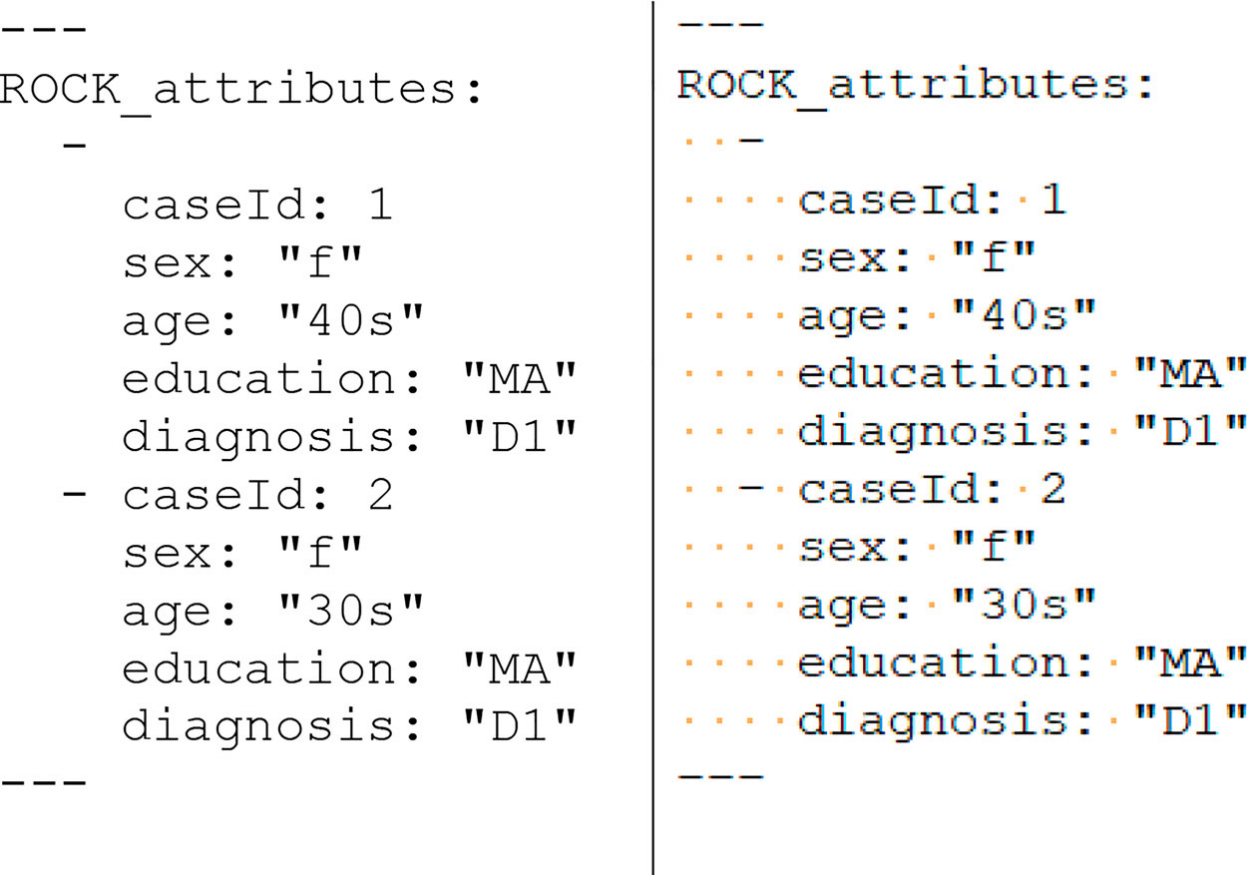
Attributes for two cases, listed in the ROCK format (left) with visible white space symbols (right).

Attributes for identifiers are specified in a YAML^2^ fragment delimited by lines containing only three dashes (“---“). Figure 4 illustrates such a fragment, populated by the attributes of interest for this tutorial for our first two data providers. By specifying categorical attributes with identical values over data providers, we can aggregate this information across cases or group utterances based on any of these attributes. Furthermore, we will be able to group and analyze utterances not only according to case, but also based on our attributes (e.g., sex, age group, education, diagnosis). Hence, for example, we can inspect all utterances by all females in the study or narratives from a certain diagnosis group. As explained above, attributes can be listed separately for each source within the source itself, or jointly in a plain text file.

### Coding and segmentation

Now that we have cleaned our sources, added unique utterance identifiers to each line of data, specified our persistent identifiers and section breaks, as well as designated our attributes, we can begin coding and segmenting our sources. The rock package facilitates automated coding (based on specific words and/or regular expressions; many other R packages exist for this purpose, too) and manual coding is possible through the iROCK graphical user interface or any plain text editor. In our worked example, we used manual coding.

#### Manual coding & segmentation with iROCK

**Figure 5.**
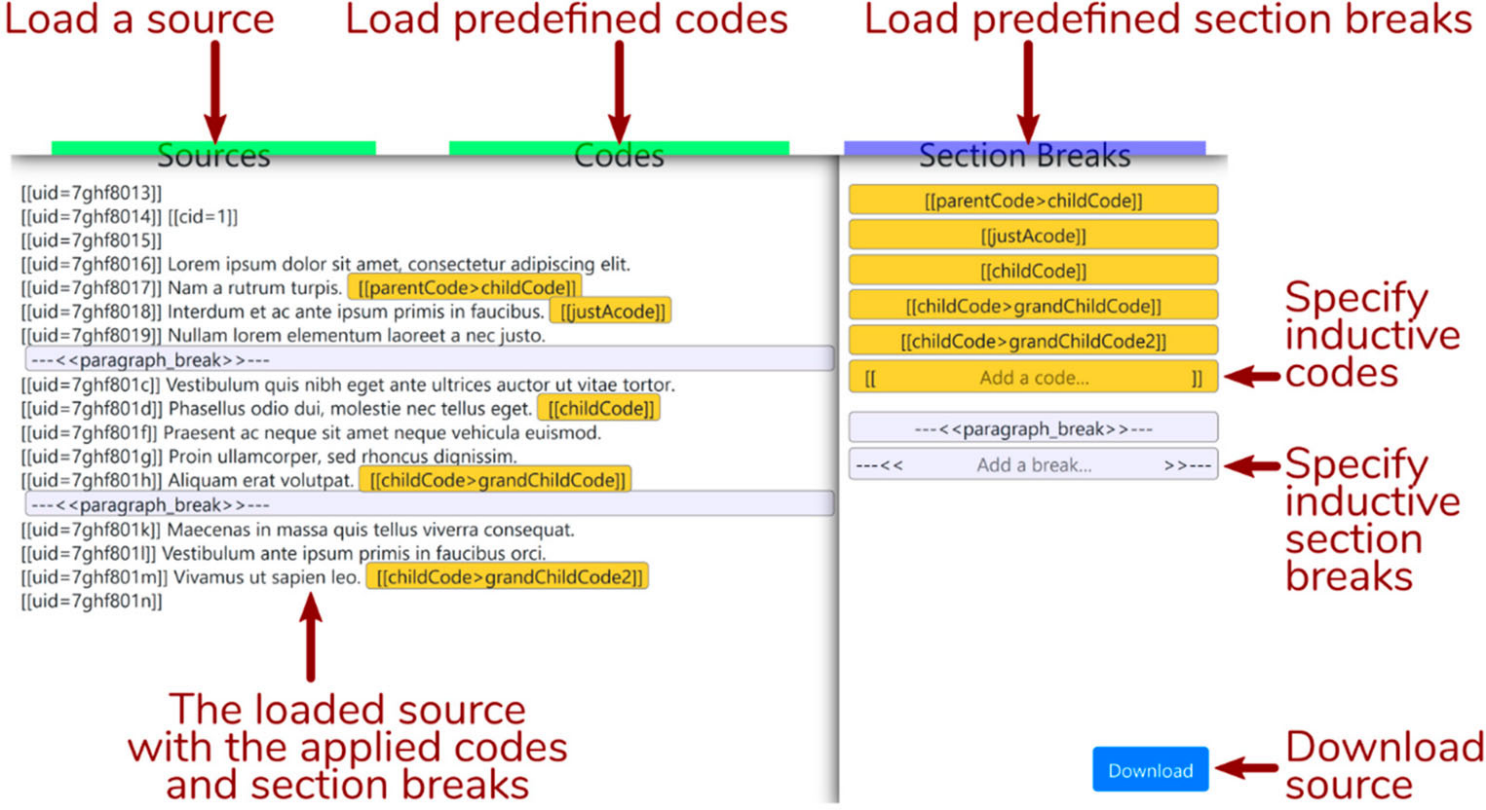
Interface for the Reproducible Open Coding Kit (iROCK).

The Interface for the Reproducible Open Coding Kit (iROCK) is a rudimentary, browser-based graphical user interface that is GDPR-compliant precisely because it runs completely in your browser: no data in the coding process is uploaded to a remote server. iROCK can be reached at http://i.rock.science; once you arrive at the interface (illustrated in Figure 5), you can load the source you wish to code by clicking on the “Sources” button (select your sources from the “030—sources-with–UIDs” directory). This action will load your source into the main pane on the left.

The interface supports both inductive and deductive coding and segmentation. To code deductively, create a plain text file containing all your code identifiers specified in the ROCK format, e.g., [[Etiology > Psych]]. Next, click on the “Codes” button in iROCK and load your codes into the pane on the right. Do the same for your section breaks (e.g., --- << stanza_delimiter >> ---), and click on the corresponding button at the top to load them into the same pane as your codes. You can drag and drop your code identifiers and section break identifiers from the pane on the right into the text. Your codes will appear at the end of each utterance (line); an utterance can be tagged with multiple codes. Your section break will be placed below the utterance on which you dropped it, between two utterances; segments delimited by various types of section breaks can overlap. If you want to code inductively, you can select the text field with the “Add a code … ” placeholder in the pane on the right, add your code identifier, hit enter, and drag and drop it on an utterance. The same procedure holds for inductively created section breaks. Once you are finished coding and segmenting your source, click on “Download” to save it to your computer. When finished, place your coded sources into the “040—coded-sources” directory. As these will be plain text files, you can load a source into iROCK as many times as you want and continue to work on it until your coding is finished.

Often, multiple coders may be working on coding data in a project. When coders are using separate codes or code systems but coding the same narratives, there may be a need to create one master file for each source that contains all codes applied by all coders. This process of merging will be addressed below, but an important data management measure in such a situation is saving each version of a source in a separate subdirectory within “040— coded-sources”. Thus, source one would have its own subdirectory containing all coded versions of source one, and so on. We recommend appending a slug (e.g., an underscore followed by the coder identifier, e.g., _coderA) to the source file names; this is to distinguish sources in this stage from those that are earlier or later and/or distinguish various versions of the source that have been coded by different coders, and it will make it easy to selectively parse a subset of sources sharing the same slug.

As an important sidenote: qualitative coding is often iterative, where until the final coding scheme is developed, many rounds of coding may take place during which codes themselves may also change. “Recoding” functions within the rock R package enable making this iterative coding journey transparent, by documenting each round of coding, as well as changes to codes. This allows you to split a code, merge codes, rename codes, add new codes, and move a child code to another parent, while documenting your justification for these actions for your future self and for other researchers.

#### Merging coded sources

**Figure 6.**
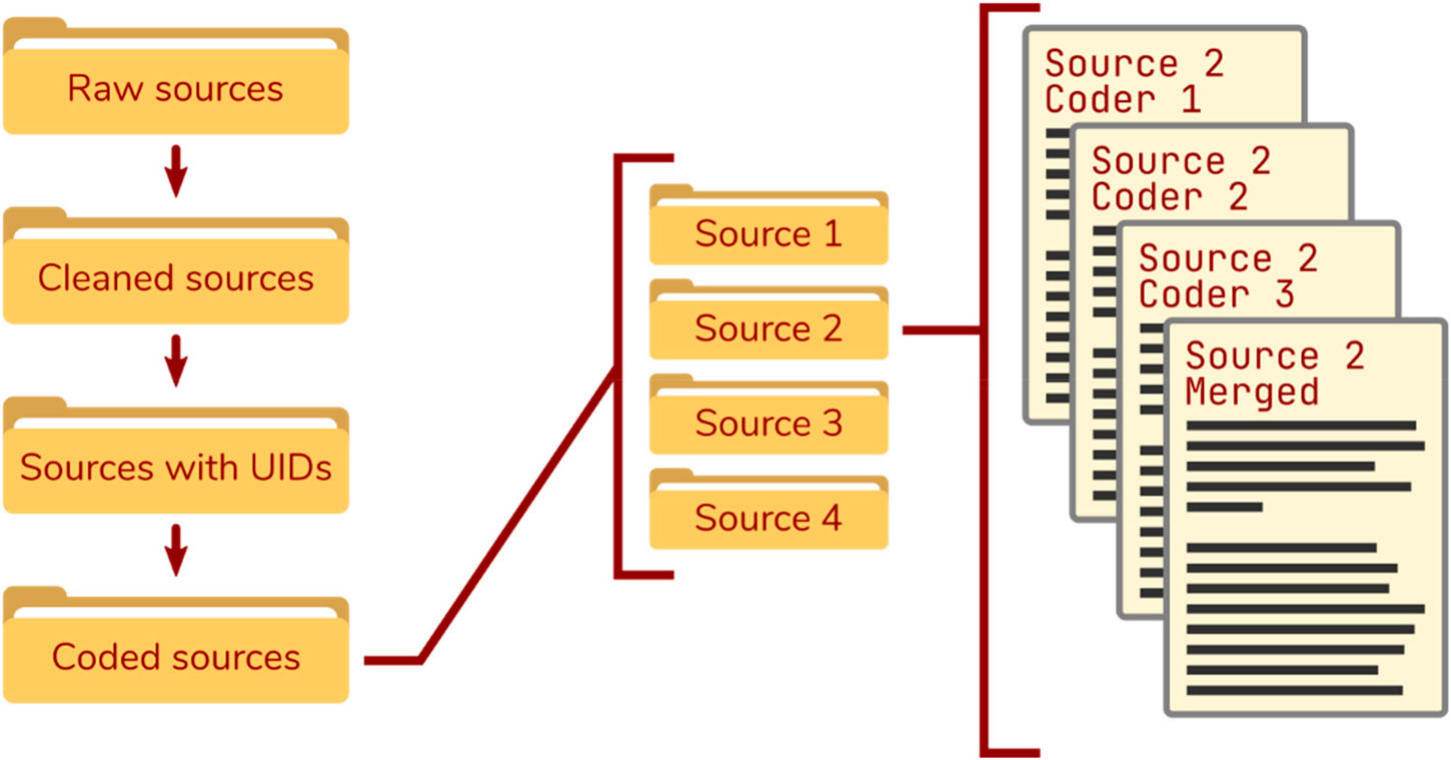
Example of the data curation and coding workflow, as well as the resulting directory structure if multiple coders are coding.

If our project necessitates a master file for each source that contains all codes by all coders, we can employ the ROCK command: rock::merge_sources(). This will create a merged file for each source and, by default, write it into the specific source’s sub-directory. By specifying a slug to append to the file name (e.g., _merged), we can keep track of which file is the merged version within each source sub-directory. Figure 6 shows the journey that our data has taken in the process of curation and coding, illustrates the structure of the “data” directory in an R project in which three coders have coded the same source (with different codes), and shows the four files that result after the three coded sources have been merged into one master file.

### Finalizing data curation and exporting

#### Overview and parsing sources

At this point in the process, we should have a set of coded and segmented (merged) sources that contain case instance identifiers, and the sources themselves contain relevant attributes or these are stored in a separate plain text file anywhere within the R project (see Figure 3 for a full illustration of what a source might look like).

In order to aggregate all the information we have added to the data and extraneous data, we can use the rock R package, specifically the command: rock::parse_sources(). This command will create a data frame containing all sources, codes, segmentation, attributes, and data.

#### Creating and exporting your data frame

A data frame constructed with the rock R package creates a “qualitative data table” (Shaffer, 2017). In such a table, utterances determine the rows, while columns represent all variables in the project. The table can contain text strings as well as categorical, numerical, and binary coding and values. Commonly, attributes are represented as categorical values, segmentation is in numerical form, and occurrence of codes (those we used to code our narratives) are indicated in binary form. Qualitative data tables also contain the raw data, one utterance per row. Utterances with case instance identifiers are supplemented with the attributes specified for those case instances, which are represented in their respective columns. Each differently identified section break is represented in a column of sequentially incrementing numbers that can be used to group together the utterances in the same segment. Codes are represented with a column each with 1s indicating that an utterance was coded with that code, and 0s indicating that it was not. Table 6 illustrates a part of the qualitative data table that would be produced when the source in Figures 3 and 5 is parsed.

**Table 6.** Excerpt of the qualitative data table generated by reading the source in Figure 3 with the rock R package.

Utterance identifiers	Clean utterances	Paragraph break counter	childCode	grandChildCode	justAcode	region	age
7ghf8016	Lorem ipsum dolor sit amet, consectetur adipiscing elit.	0	0	0	0	urban	60s
7ghf8017	Nam a rutrum turpis.	0	1	0	0	urban	60s
7ghf8018	Interdum et ac ante ipsum primis in faucibus.	0	0	0	1	urban	60s
7ghf8019	Nullam lorem elementum laoreet a nec justo.	0	0	0	0	urban	60s
7ghf801c	Vestibulum quis nibh eget ante ultrices auctor ut vitae tortor.	1	0	0	0	urban	60s
7ghf801d	Phasellus odio dui, molestie nec tellus eget.	1	1	0	0	urban	60s
7ghf801f	Praesent ac neque sit amet neque vehicula euismod.	1	0	0	0	urban	60s
7ghf801g	Proin ullamcorper, sed rhoncus dignissim.	1	0	0	0	urban	60s
7ghf801h	Aliquam erat volutpat.	1	0	1	0	urban	60s
7ghf801k	Maecenas in massa quis tellus viverra consequat.	2	0	0	0	urban	60s
7ghf801l	Vestibulum ante ipsum primis in faucibus orci.	2	0	0	0	urban	60s
7ghf801m	Vivamus ut sapien leo.	2	0	0	0	urban	60s

To ease flexible representation and interchangeability, the rock command rock::expand_attributes() can be employed to expand a categorical variable into a series of dichotomous variables (e.g., expanding attribute “age” with categories “40s”, “50s” and “60s” results in three new binary columns; “age_40s”, “age_50s”, and “age_60s”).

We may want to export our data frame to a spreadsheet, .csv file, or SPSS data set for further processing. We can do this with the “rock::export_mergedSourceDf_to_*()” function family (e.g., “rock::export_mergedSourceDf_to_csv()”). Provided we want to generate epistemic networks, this exported object can be uploaded to the ENA webtool (although the rENA R package also allows directly applying ENA).

### Generating network graphs

#### Constructing epistemic networks

Describing in detail how networks are computed and generated does not fall within the scope of this tutorial; ENA as a data modeling method is described elsewhere (Bowman et al., 2021; Shaffer, 2017). Succinctly, for each stanza (set of one or more utterances; e.g., responses to an interview question), ENA produces a matrix with code co-occurrences and calculates the co-occurrence frequency of each unique pair of codes within that given segment of data. Following this, ENA computes the cumulative frequencies for each unit of analysis; usually units are defined as data providers or groups of data providers. The cumulative co-occurrence matrix for each unit is represented as a vector, and the vectors for all units together can be considered to form an *n*-dimensional space. Due to the fact that these vectors may vary in length (because they contain co-occurrences from different amounts of qualitative data), each unit’s vector is divided by its length. This normalization of vector lengths captures the relative frequencies of code co-occurrences independent of discourse length. Subsequently, ENA applies singular value decomposition (SVD), a dimensional reduction procedure similar to principal components analysis, to reduce the *n* dimensions to two dimensions. These two dimensions form the axes along which the unit vectors (containing the co-occurrences aggregated for each unit) are then projected as points into a two-dimensional space. These are referred to as plotted points (or ENA scores).

The SVD thus produces a two-dimensional space in which the ENA scores can be plotted. To facilitate interpretation of the locations of the ENA scores in this reduced space, ENA uses an optimization routine to position the nodes of the networks in the reduced space such that for any unit, the centroid of its network graph is as close as possible to the location of its plotted point. This is done using a least-squares method, and the goodness of fit can be measured by computing the correlation (e.g., Pearson's or Spearman's r) between the ENA scores and the corresponding centroids on each dimension. The positions of the nodes are in the same place for every network, and so the node positions can be used to interpret the dimensions and the locations of the networks on those dimensions. This also facilitates comparison of networks both visually and via summary statistics.

Thus, ENA offers two coordinated representations of the data: 1) Plotted points, or the position of each network in the two-dimensional space, and 2) Network graphs per unit in which the nodes represent the codes and the edges depict how the relative frequency with which each pair of codes co-occurred within the specified segments of data. The coordination of network graphs and plotted points means that the positions of the nodes can be used to interpret the dimensions forming the constructed space and explain the positions of plotted points. The *x* axis represents the SVD dimension that explains the most variation in the co-occurrences, while the *y* axis represents the SVD dimension that explains the most variance in the co-occurrences after the variance explained by the first dimension has been partialled out. Networks may also be compared using network difference graphs (comparison plots). These graphs are calculated by subtracting the weight of each connection in one network from the corresponding connections in another.

When only two units are compared (such as, in our example, the biomedicine group and the CAM group), the first dimension (mapped onto the *x* axis) can be constructed with a procedure called means rotation, which first rotates the *n*-dimensional space to maximize the distance between the mean networks of the two units. The second dimension (mapped onto the *y* axis) is determined by performing an SVD to explain the remaining variance.

#### Key model parameters

As mentioned, ENA models are generated for units of analysis, which can be operationalized in a variety of ways even within a single project. A unit is whatever or whoever we would like to see a network for: various groupings of data providers or individual cases are commonly designated as units of ENA graphs. In our example results above, we chose to define units as Group (biomedicine versus CAM users) and Source (which in our study was the same as case, that is, individual data providers). We chose this so that we could model the mean networks of the two groups, but also to inspect the ENA scores (and network graphs) of individual cases within the generated projection space (see Figure 1). We also chose to create networks for biomedicine versus CAM users in three diagnosis groups (see Figure 2). In this instance, our units were selected as Sources within Diagnoses within Groups (styled as “Group > Diagnosis > Source” in ENA). Operationalizing units in such a nested way enables us to inspect the mean networks for our subsamples (Biomedicine versus CAM), groups within those (diagnosis groups D1-3), and individual networks for our cases.

Since code co-occurrences are affected by the length of talk (i.e., groups of utterances, segments) in which they are allowed to interact, segmentation and defining relational context (grouping of utterances which provides meaning for each line of data) are crucial steps in the modeling process. ENA uses two parameters to operationalize relational context: conversation and stanza window. Conversations are groupings of utterances that can be connected in a model; we chose Source as our conversation, as we only wanted to allow code co-occurrences to take place within the same interview transcript and not across various transcripts. We could also use Source > Stanza as our conversation if we wanted to use our stanzas (designated by “--- << stanza_delimiter >> ---“) to provide tighter relational context. These decisions would be based on our research question, the codes under scrutiny, and our theory of how and under which conditions code co-occurrence would be indicative of the phenomena we are interested in, for example, psychological proximity of the coded concepts.

Relational context is also critically determined by stanza windows. A stanza window is a mode of co-occurrence accumulation, a specific way of operationalizing stanza. There are several types of stanza windows; the decision to use either is based on assumptions regarding relational context, code positions within the source and relative to each other, and whether a weighted or unweighted network model is desired. Stanza window types, sizes, and their effects on network models are described elsewhere in greater detail (Siebert-Evenstone et al., 2017; Zörgő, Swiecki, et al., 2021). In sum, data is coded on the level of utterances, while stanza and conversation provide relational context for utterances. Code co-occurrences are accumulated via stanza windows and are aggregated on the level of conversation for each unit of analysis.

### Strengths of Epistemic Network Analysis

#### Opening the black box of hermeneutics

Many scholars have argued that qualitative research often entails an analytical process similar to a black box: hermeneutic processes in qualitative research are usually not explicit, thus making it difficult to ensure transparency in how results were produced (Shelton et al., 2014). This can also create a gap between real-world insights and transferrable outcomes (ibid). Through the systematic coding of qualitative data, the process of interpretation can (to some extent) be made transparent. For example, manually applying each instance of a code requires careful consideration by the researcher. In this process, coding decisions can be documented and justified, and subsequently can be published transparently. ENA forms an additional lens on your data, allowing its qualitative and quantitative aspects to inform each other, and as such, provides an additional warrant for your claims.

#### Aiding reflexivity

Albeit reflexivity is a central practice to qualitative research, it is defined in a myriad of ways and can often remain ambiguous in its practical application. Some scholars see reflexivity as a way to control (or at least become conscious of) bias, while others emphasize introspective and intersubjective reflections between researcher and data provider. (Buetow, 2019; Morse et al., 2002) Epistemic networks can serve as a tool in reflexivity with which the researcher may iterate between the raw (or coded) data and its statistical model to identify their own (preliminary) assumptions, alternative or rivaling interpretations, and contextualized code interactions, thus “closing the interpretive loop” (Shaffer, 2017). Both the ROCK and ENA allow direct access to the coded data within the user interface.

#### Moving beyond code frequency

We could examine code frequencies or relative code frequencies (normalized to account for the differing lengths of narratives) to answer our research question: Are there any differences between how biomedicine versus CAM users conceptualize etiology of illness, and if so, what are these differences? With such a technique, we may observe that psychosocial factors were the most prevalent etiological code, while ecological factors were the least mentioned. Yet, since etiologies are multifactorial (Helman, 1994) this would not help us map what elements compose an etiology or understand their interplay. By displaying various etiologies as network nodes and depicting their interplay as edges, ENA can also show us that while Biomedicine users emphasized a combination of psychosocial, genetic, and ecological factors, CAM users most often spoke of a constellation of psychosocial, vitalistic, and nutritional factors. Because we have direct access to the coded data, we can also examine what these edges (co-occurrences) mean; this is crucial to answer *how* Biomedical and CAM lay etiologies differ. By scrutinizing network edges, we can observe that vitalism is connected to all other nodes, serving as “prism” through which the patient views each etiological factor. For example, genetic factors co-occurring with vitalism may connote an illness occurring as “karmic punishment”. Please see Zörgő, Peters, et al. (2021) for more examples comparing code frequency counts versus epistemic networks with this specific data.

#### Conveying complexity

A common problem qualitative researchers face in presentations and write-ups of their work is the lack of time and space to expound the complexities of their findings. ENA offers a visualization that aids conveying such complexity in a way that caters to both qualitative and quantitative sensibilities. Visualizations are known to boost interpretability and improve recall (Larson, 2009).

### Caveats and limitations

#### Requires theory of co-occurrence meaning

One important consideration when using ENA is that the researcher must have a clear theory that links code co-occurrences in segments of the used size to a meaningful theoretical construct. A priori, there is no reason to assume that code co-occurrence has any epistemic meaning; this epistemic inference must come from auxiliary assumptions. If a researcher cannot formulate a model that explains what can be learned from (absence of) patterns in co-occurrence frequency, the “Epistemic” in ENA rings hollow. However, note that the same is true for other analytical approaches to qualitative data. Inferences from attached codes always require auxiliary assumptions (e.g., some introspective ability on the part of the participants; ability to express mental processes, feelings, and thoughts without verbalization introducing bias; etc.).

#### No hypergraph capabilities

At the time of writing this tutorial, ENA does not yet support hypergraphs. In other words, it only supports edges from one code to another (i.e., 1:1 relationships between codes), whereas co-occurrences potentially can span multiple codes simultaneously (i.e., n:n relationships between codes).

#### Central role of segmentation

Because in ENA, segments play a large role in establishing the networks, if the segmentation changes, the results are likely to change. This places segment definitions and segmentation procedures at the heart of the analysis. These far-reaching effects of segmentation decisions are not immediately obvious (as the codes, not the segments, often most readily correspond to the phenomena of interest). In addition, developing segment definitions that yield clear-cut and unequivocal mutually exclusive segments can be very challenging.

## Conclusions

In this tutorial we have introduced Epistemic Network Analysis (ENA). ENA provides a unique perspective on a qualitative data set as a way to shed new light on the studied phenomena. It leverages the qualitative data table, a product of the unification of qualitative and quantitative methods afforded by segmenting qualitative data and quantifying the applied codes at the level of the smallest codable segment. We have also shown how the Reproducible Open Coding Kit (ROCK) provides a way to accessibly and transparently document the process from raw data files to coded versions, and have illustrated how the rock R package facilitates implementing this. We have explained how to produce ENA plots using such qualitative data tables and shown how these may be interpreted.

By providing a novel lens through which to view qualitative data, ENA can aid in obtaining new perspectives and better grasping the phenomena under study. Sometimes, ENA can even serve a direct inferential purpose, specifically when proximal code co-occurrence can reasonably be considered as representing specific aspects of the topic of interest. However, as tempting as it may be to interpret the edges as indicative of connections between the concepts represented by the codes connected by those edges in general, this inference is not necessarily valid. Co-occurrences may be just that: co-occurrences; and simultaneously coding a data fragment with two concepts does not necessarily allow meaningful inferences as to the research question.

As such, ENA can be considered to have two general applications. First, there is the descriptive case, where visualizing the patterns in the data as manifest in the co-occurrences of codes affords a new perspective on the phenomenon under study. Second, there is the more fundamental use in scenarios where there is a theory as to why code co-occurrences are directly indicative of one or more aspects of those phenomena under study, such as psychological proximity of the concepts represented by the codes.

In both situations, ENA allows health psychologists to obtain insights otherwise unavailable by efficiently presenting relative code frequencies and co-occurrence patterns and facilitating comparison of those patterns, both between groups and for individual data providers. We hope that this tutorial makes this method accessible to the wider community of health psychologists and other scholars.

### Abbreviations and terms

Attribute: Characteristic of data, data provider, or aspect of the study process

CAM: Complementary and Alternative Medicine

Case: Data provider; a type of class instance

Class: Organizational principle used to encode data provenance (e.g. “participants” or “locations”) or process (e.g. “coder”)

Class Instance: an instance of a class (e.g. “participant 5” or “the library”), used to attach attributes to utterances

Class Instance identifier: brief identifier to uniquely refer to a class instance (e.g. “5” or “library”)

CLIFF: Clean Lean Initial Formatted File

Conversation: ENA model parameter operationalizing relational context; codes are only allowed to co-occur in this grouping of data

Code: Concept relevant to the research question that is used to label fragments of qualitative data

Code identifier: Unique identifier that, when appended to utterances, attaches the corresponding code to that utterance

Data frame: Data structure in R; rectangular list of vectors organized in columns

Hierarchy marker: The greater-than symbol, that the ROCK uses to indicate parent-child relationships between code identifiers. For example, “someCode > itsChild > itsGrandchild” represents a structure with three levels where someCode is closest to the root. If one of the parsed sources also contains “someCode > siblingCode”, in the merged code tree the code identified by “someCode” would have two child codes, one identified by “itsChild” and one identifier by “siblingCode”, and the first of those would have a child code of its own, identified by “itsGrandchild”.

ENA: Epistemic Network Analysis

Identifier: a symbol formed by a unique string of characters to identify a code, type of section break, or class instance. In the ROCK standard, identifiers must all begin with a lowercase or uppercase Latin letter (a-z or A-Z) and may only contain Latin letters, Arabic numerals (0–9), and underscores (_). Examples of identifiers are “someCode”, “turn_of_talk”, and “participant_52”.

iROCK: Interface for the Reproducible Open Coding Kit

ROCK: Reproducible Open Coding Kit

Section break: A delimiter used to segment data

Section break identifier: Unique identifier inserted between utterances when segmenting data

Segmentation: Dividing the data into mutually exclusive fragments that are consistent with the segment definition

Source: File containing (potentially coded) qualitative data

Stanza: Form of higher-level segmentation; grouping of utterances

Stanza window: ENA model parameter for co-occurrence accumulation

SVD: Singular Value Decomposition

Unit: ENA model parameter; code co-occurrences within stanzas are aggregated within units

Utterance: Segment defining the smallest codable unit of data

YAML: human- and machine-readable data-serialization language; the recursive acronym stands for “YAML ain’t markup language”

## Notes

1 This shorthand, “cid”, refers to “caseId”, and is specified in another setting for the rock package (“idRegexes”; use ?rock::opts for an overview of the options)

2 YAML is a human- and machine-readable data-serialization language; the recursive acronym stands for “YAML ain’t markup language”
